# Excellence fulfilled? On the unique developmental needs of professional athletes

**DOI:** 10.3389/fspor.2023.1164508

**Published:** 2023-04-25

**Authors:** Joseph Baker, Kathryn Johnston, Harjiv Singh, Lou Farah, Dale Lablans

**Affiliations:** ^1^ School of Kinesiology and Health Science, York University, Toronto, ON, Canada; ^2^ Department of Kinesiology and Nutrition Sciences, University of Nevada, Las Vegas, NV, United States

**Keywords:** athlete development, player longevity, sport administration, career transitions, talent development, talent retention

## Abstract

While the term “athlete development” has been used to capture the changes (physical, psychological, etc.) that occur as an athlete moves from initial sport engagement to elite performance, much of the research in this area has focused on earlier stages of the pathway, with very little work examining the highest levels of sport. Considering a person's bio-psycho-social development continues through adulthood, the limited attention to development for athletes at higher competitive levels is perhaps surprising. In this short article, we highlight several notable discrepancies between different competitive levels (e.g., pre-professional sport and professional sport) in the way development is conceptualized, contextualized, and operationalized. We use available evidence to provide guidance for researchers and practitioners to encourage the delivery of structured developmental programming in professional sport systems to aid with the transitionary period between pre-elite and elite levels, and to help foster career longevity.

## Key points

• Little is known about how to support athlete development at the highest levels of sport participation
• As athletes continue to develop and evolve, structured developmental programming is necessary to support athlete transitions, career longevity, and the overall athlete experience

## Introduction

The sudden retirement announcement of the highest ranked female tennis player in the Women's Tennis Association in 2022, Ash Barty, came as a shock to many. Questions such as “*what led to this decision?”* and “*how could this have been prevented?”*, drew attention to the limits of our understanding regarding athlete wellbeing, along with support and development for athletes at the professional level. Just as there are unique elements that relate to the quality of the coaching and learning environments during early development (e.g., we do not train youth as if they are mini adults), there are almost certainly factors related to later phases of development that underpin an athlete's capacity to thrive (i.e., balancing one's psychological, interpersonal, and physical resources) during this time. In this short piece, we argue that research initiatives and structured programming for athlete development are needed at the professional sport level, arguably with the same rigor and importance as pre-professional sport. Identifying and optimizing these factors could have important implications for wellbeing and performance at this level of athlete development – which may ultimately prevent unfortunate events such as the one had by Ash Barty.

It is important to note that the term “development” has multiple definitions, varying in context and priorities, but generally it relates to the process of growth, change, and stabilization across the lifespan. In the context of education, development is closely tied with learning. Although there is much discussion amongst scholars in the field of education about whether the environment should be formally (i.e., organized and structured) or informally (i.e., more happenstance) designed to maximize learning, nearly all models of learning and development propose a crucial role for the environment in influencing the type and quality of development that occurs. While there is value in exploring the impact of each of these components of the learning environment as it relates to sport, in this paper, we focus on “organized interventions” (1) and “deliberate programming” (2); that is, the formal and explicit environments that are designed to improve an athlete's development in some sense (e.g., physically, physiologically, emotionally, and/or psychologically).

This paper's authorship includes researchers and practitioners in various disciplines of sport science and professional practice, who together have recognized a deficit in peer reviewed research, coupled with a lackluster delivery of athlete developmental programs/initiatives with professional teams. To be fair, some teams do this better than others, but, as a whole, these initiatives are applied inconsistently at this level for a multitude of reasons (i.e., financial resources, interest, time, etc.). This paper draws together our perspectives on the matter framed against current evidence, to provide suggestions for researchers and practitioners on “*why*” and “*how*” to enhance the delivery of such programs. While we generally focus on North American professional sports (primarily the National Basketball Association, National Hockey League and Major League Baseball), many of the issues apply more generally in sport systems from other parts of the world.

### Development in the context of professional sport

For athletes who make it to the professional level, most (if not all) have spent considerable time (i.e., years or even decades) in high-performance (i.e., excellence-driven) development systems. Along this pre-professional journey, athletes are typically provided opportunities to focus on developing the wide range of technical, tactical, physical and mental/psychological skills needed for exceptional performance in their sport. This is often embedded into the fabric of the sport program, and formally and informally woven into training and practice sessions.

One notable difference, however, between the pre-professional system and the professional system relates to the discrepancies in how “development” is prioritized. At the professional level, it appears (at least on the surface), that the primary outcome of interest shifts away from “development” and towards “maximizing performance”. This may involve, for example, fine tuning aspects of injury mitigation (e.g., the increased use of strength and conditioning or focused recovery/medical practices) or tactical improvement (e.g., learning or modifying game strategies). Of course, performance coaches may also aim to improve and develop performance-related skills (e.g., improving a free throw in basketball or a pitcher's throw in baseball), but it seems that athletes are seen as more “fully formed entities” at the professional level than they would at lower levels of skill. In many ways they are (e.g., they would be expected to have a solid grasp of most of the “fundamentals” of their sport); however, there may be critical areas of personal and professional development that are neglected in many professional sport environments in North America.

### Unique demands at the professional level

As suggested above, there may be unique developmental demands related to the professional environment. For instance, professional sports often have condensed travel schedules (e.g., 41 regular-season away games in a span of 6 months in the NBA and NHL) compared to the schedule of the amateur athletes in the same sport (e.g., amateurs usually have greater instability in their schedules with fewer competitions). These travel demands may limit opportunities for continued skill development for athletes and coaches. For instance, a recent development in some professional teams is “micro-dosing sessions” for the purpose of motor skill development, to accommodate the infrequency of an athlete being at their home training facility. Furthermore, travel demands may also increase an athlete's physical and mental load (i.e., volume of demands) while compromising approaches to recovery and learning (e.g., increasing the frequency of disruptions in sleep patterns), not to mention how it may affect their social and personal life (interactions with family and friends, caretaking, etc.). Relatedly, competition schedules may affect the types of performance-related priorities athletes (and teams) focus on. For example, a heavier game and travel schedule may result in athletes losing weight during the season. As a result, the focus for athletes and their support teams (e.g., sport medicine team) becomes maintaining muscle mass and injury prevention. At lower levels of competition, when athletes are less physically mature, there may be a bigger emphasis on gaining muscle mass and developing in ways that increase the likelihood of future success.

Differences in psycho-social demands are also important to consider. At the professional level, there are unique pressures on athletes that may be less prevalent in pre-professional or amateur sport. One of which is monetary pressure. In this sense, young players entering professional leagues are incentivized to progress in such a way that guarantees them a roster spot on the first team where they can make ten times (or greater) what their salary would be if they play in the “minor leagues”. This adds an extra layer of pressure that collegiate or Olympic athletes, for instance, do not experience to the same extent. Of course, at later stages of athlete development, all individuals still in the system may navigate the possibility of developing into professionals in order to gain financial rewards, but this pressure is maximized in professional sports. More specifically, players can lose their spot on their team’s roster, and large proportions, if not all, of the associated monetary rewards, on any given day. Closely connected to this change in earning is the fiscal responsibility that comes along with it. Sadly, there are numerous stories of professional athletes who sign significant contracts only to eventually find themselves in large debts (3, 4).

### Challenges with developmental programming at the professional level

While these developmental differences between the levels of sport make intuitive sense, the reality is, there is little empirical evidence exploring these observations. Despite the importance of, and need for, this work at the professional level, improving understanding in this area will be difficult - undoubtedly because this type of work requires high-quality, longitudinal approaches. This type of research is challenging to do at the professional level for several reasons including confusing/conflicting terminology, regular movement of players from one team (i.e., developmental environment) to another, the influence of acute and chronic injuries, as well as privacy and confidentiality concerns with player data at this level.

These challenges will be hard to overcome for several reasons. Methodologically, factors related to professional athlete development are difficult to measure in valid and reliable ways (at least at the time of this article). Moreover, gaining access to these populations is difficult. Often there are strict confidentiality regulations in place to avoid “secret sharing” and to maintain competitive advancements (i.e., training techniques, within team research advances). Another challenge that is unique to professional settings is the difficulty in measuring the development and growth of players in such a homogenous pool, as well as different types of proxies that could be used to indicate progress. For instance, at junior levels of competition, progress can be assessed using performance benchmarks or tracking players who separate themselves from their peers by making to the next level. However, at the highest level of competition, where improvements in various facets of performance are more incremental, tracking and quantifying such progress is more difficult. Moreover, at this stage of skill, regular assessments to evaluate athletes' potential for success at the “next level” are typically over, meaning regular performance tests that extend beyond “performance” (i.e., their actual performance in their sport measured by metrics on the ice, field, court, etc.) are limited. This leaves a gap in understanding how progression occurs at the highest levels.

### Overcoming obstacles: A call to action

Despite the challenging work ahead, solutions and evidence-informed programming may be within reach with appropriate effort. However, our optimism is bolstered by several factors. First, the funding available in professional sport for maximizing player development is impressive and may be accessible if appropriate links can be made between improving player development (and presumably indicators of health, wellbeing, and skill acquisition) and indicators with the greatest relevance to the team (e.g., improvements in competitive performance and/or returns on investment by developing players who are “in house” more efficiently). Second, the rapid growth in support team members with “development” in their job title emphasizes the latent potential already in the system. What is needed, however, is greater clarity on the priorities of such “development”, but also training and education for those in that role to support such practices. This could include continued education for staff members including internal and external courses and workshops, focused on delivering high quality and evidence-informed practices for athletes. Finally, the interaction between scientific researchers and professional teams has increased considerably in the last decade (e.g., researchers are sometimes embedded into the staffing model), and this can offer access to domain-specific expertise, at a time when research is becoming more sophisticated and difficult to translate into practice, all while promoting efficiency in time and learning resources. Collectively, these factors suggest the time is ripe for meaningful work in this area.

**Figure 1:**
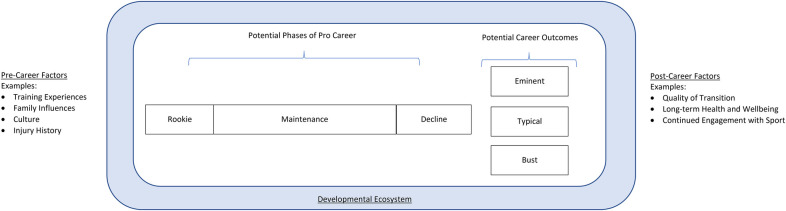
A proposed model of stages of a professional career. Each stage (i.e., initiation, maintenance, decline) will have specific goals and objectives that link to different performance and development outcomes. Furthermore, stages will be influenced by pre-career factors and the developmental ecosystem surrounding the athlete (e.g., organizational priorities, quality and nature of support system and resources, training and education available for those in developmental roles). Effective management of these objectives will influence post-career factors.

Based on the limited prior work in this area, we propose a crude pathway for considering how players might develop during their professional careers and what might influence this development (Figure 1). In this pathway, players move through career stages (from rookie season to eventual performance decline), with each stage differing relative to what is occurring in an athlete's development during this time. For instance, athletes entering the professional league may need assistance making the transition from college or high-school levels into the new environment (5, 6). Ultimately, players' experiences in the league have the potential to lead to one of three outcomes. The majority will leave the league within the first year, while the majority of those who stay in the league have typical careers generally lasting on average between 5.5 years (for National Football League players) and 8.2 years (for NBA players) (7). A very small minority will go on to have “eminent” careers, ending up in the Hall of Fame or winning Most Valuable Player awards (8). Understanding how to optimize players' movements through these stages of a professional career, as they relate to predicting these outcomes, remains an important area of future research.

Three areas of future research are suggested. The first is to explore *how* elements of development change across an athlete's professional career. Based on prior work on stages and phases of athlete development, it seems reasonable to assume there are qualitatively different “phases” across a professional athlete's career. These phases would be defined by changes in the weight/value of different developmental priorities. For instance, early in an athlete's time at the professional level it may be more critical to focus on helping them make the transition from “high potential amateur” to “proving your potential rookie”. We know very little about the psychological dynamics occurring during the period, but it is likely they are highly nuanced and complicated. Moreover, we know little about how the environment of professional sport constrains this development (e.g., a rookie drafted to a stronger team may receive less playing time than one drafted to a weaker team, which may affect opportunities for development). After making this transition to the professional level, players may go through other phases as they attempt to maintain high levels of performance before the inevitable performance decline that leads to retirement and transition out of the professional system.

A second area to explore is the *types* of elements affecting player development outcomes at the professional level. Apart from a plethora of studies on the influence of various injuries (9, 10), few studies (7, 11) have examined predictors of career length. Even within these studies, the “success” of a professional athlete's career is captured using simple metrics (i.e., how long was their career?). Moreover, emerging concepts such as sporting “eminence” (8, 12) indicate that in addition to different phases across professional development, there are different outcomes for career achievement to be considered (e.g., was the athlete an “average” pro or an eminent one?).

Third, an obvious and important direction for future research is to examine the programmatic factors that predict these different metrics of career success. For instance, “player development” is perhaps the most rapidly growing category of personnel within professional sport systems. Understanding the relationships between different elements of the system, including the roles and responsibilities of different program staff, could provide insight that drives more efficient, cost-effective systems while improving player health, welfare, and performance. Moreover, understanding player needs across these crude stages (e.g., how they change over time and why) and exploring the importance of different categories of variables within each stage (e.g., performance-related changes vs. changes in social or psychological variables), may provide important indicators of player wellbeing.

### Concluding thoughts

Research examining ways to promote and implement developmental programming for professional athletes remains scarce. What is clear, is that the ability to consistently demonstrate exceptional performance at this level reflects a complicated process of continued skill development, maintenance or improvements in strength and conditioning, and sustained general physical and mental health, usually alongside significant changes in life circumstances. For this reason, more work is needed to promote an increased understanding of ways to engage and support athletes across their entire sporting journey, along with ways to improve wellbeing, enhance sport experiences, and increase career longevity. In an era where professional athletes are known for their constant focus on performance in their sport, the news of Ash Barty highlights the complexity in balancing organizational success with an individual's expectations for success. In an effort to address the issues in the wake of Ash Barty's retirement and echoing her sentiments of, “I know how much work it takes to bring the best out of yourself. And…I don't have that in me anymore…” re-thinking development is a critical endeavor for the future of professional sport (13).

## Data Availability

The original contributions presented in the study are included in the article, further inquiries can be directed to the corresponding author.
